# Combined Use of cyclinD1 and Ki67 for Prognosis of Luminal-Like Breast Cancer Patients

**DOI:** 10.3389/fonc.2021.737794

**Published:** 2021-11-09

**Authors:** Junmei Hao, Wenfeng Zhang, Yan Lyu, Jiarui Zou, Yunyun Zhang, Jiahong Lyu, Jianbo Zhang, Shuishan Xie, Cuiping Zhang, Jiandi Zhang, Fangrong Tang

**Affiliations:** ^1^ Department of Pathology, Yantai Affiliated Hospital of Binzhou Medical University, Yantai, China; ^2^ Yantai Quanticision Diagnostics, Inc., Yantai, China

**Keywords:** QDB, FFPE, Ki67, Luminal-like, breast cancer, cyclin D1

## Abstract

**Background:**

Ki67 is a biomarker of proliferation to be used in immunohistochemistry (IHC)-based surrogate assay to determine the necessity of cytotoxic therapy for Luminal-like breast cancer patients. cyclinD1 is another frequently used biomarker of proliferation. A retrospective study was performed here to investigate if these two biomarkers may be combined to improve the prognosis of Luminal-like patients.

**Methods:**

Both Ki67 and cyclinD1 protein levels were measured absolutely and quantitatively using Quantitative Dot Blot method in 143 Luminal-like specimens. Optimized cutoffs for these two biomarkers were developed to evaluate their prognostic roles using Kaplan–Meier overall survival (OS) analysis.

**Results:**

cyclinD1 was found as an independent prognostic factor from Ki67 in univariate and multivariate OS analyses. At optimized cutoffs (cyclinD1 at 0.44 μmol/g and Ki67 at 2.31 nmol/g), the subgroup with both biomarkers below the cutoffs (*n* = 65) had 10-year survival probability at 90% in comparison to those with both biomarkers above the cutoffs (*n* = 18) with 8-year survival probability at 26% (log-rank test, *p <*0.0001). This finding was used to modify the surrogate assay using IHC-based cyclinD1 scores, with *p*-value decreased from 0.031 to 0.00061 or from 0.1 to 0.02, when the Ki67 score of 14 or 20% was used as cutoff, respectively, in the surrogate assay.

**Conclusion:**

The current study supports the prospective investigation of cyclinD1 relevance in the clinic.

## Introduction

The Luminal-like breast cancer, featured by the overexpression of estrogen receptor (ER), is defined in immunohistochemistry (IHC)-based surrogate assay as tumors with ≥1% positive staining of ER in daily clinical practice (1–4). This group of tumors is further separated into Luminal A-like and B-like subtypes based on a protein biomarker of cell proliferation, Ki67. Based on the St. Gallen consensus issued in 2013, those tumors with Ki67 <14%, progesterone receptor (PR) ≥20% based on IHC analysis, and human epidermal growth factor receptor (Her2)-negative (Her2-) are defined as Luminal A-like subtype with better prognosis. The rest of the Luminal-like tumors are classified as Luminal B-like subtype (2). In 2015, the cutoff for the Ki67 score to distinguish Luminal A-like from Luminal B-like patients was adjusted to 20% (5). While multi-gene testing remains inaccessible to many patients worldwide, the IHC-based surrogate assay becomes the primary tool to determine the necessity of cytotoxic therapy for Luminal-like patients in daily clinical practice (2, 5).

cyclinD1 is another commonly used protein biomarker of cell proliferation in routine clinical practice. While the exact biological role of Ki67 remains unclear until now (6), cyclinD1 is known as the key regulator of cell cycle progression. The overexpression of cyclinD1 drives the cells from G1 to S phase in mitosis (7). In this study, we set out to investigate if there were any prognostic differences between these two protein biomarkers of proliferation and, more importantly, if we can improve the prognosis of Luminal-like patients by using both protein biomarkers simultaneously.

In a previous study, we measured the Ki67 protein levels absolutely and quantitatively in 155 Luminal-like formalin-fixed paraffin-embedded (FFPE) specimens using our independently developed Quantitative Dot Blot (QDB) method (8). When replacing IHC-based Ki67 scores with absolutely quantitated Ki67 levels, we found that the prognosis of surrogate assay was improved significantly for the overall survival (OS) of Luminal-like tumors, with 10-year survival probability (10y SP) for Luminal A-like patients at 91 *vs*. 63% for Luminal B-like patients (8). In comparison, the 10y SP for Luminal A and B-like group was 88% and 68%, respectively, when using a Ki67 score of 14% as cutoff based on the 2013 St. Gallen consensus (2), and 84% and 70%, respectively, when 20% was used as cutoff based on the 2015 St. Gallen consensus (5).

Along this line of thinking, in this study, the cyclinD1 protein levels were also measured quantitatively and absolutely using the QDB method in the same 143 Luminal-like FFPE specimens reported in the previous study, and we used these results to demonstrate that cyclinD1 was an independent prognostic factor from Ki67. The combined use of these two protein biomarkers promises to improve the prognosis of Luminal-like tumors.

## Materials and Methods

### Human Subjects and Human Cell Lines

The inclusion criteria for this retrospective observational study included patients diagnosed with breast cancer, with FFPE tissue specimens available at Yantai Affiliated Hospital of Binzhou Medical University, Yantai, China, from 2008 to 2013 consecutively and non-selectively. The specimen must have more than 50% tumor tissue based on H&E staining. All the treatments were adjuvant treatment. Follow-up data were available for 143 patients out of 155 Luminal-like patients (92.3%), with the last follow-up on April 1, 2019. The endpoint of the overall survival analysis was defined as the time between breast cancer surgery and death or last follow-up (April 1, 2019). The median time to censoring was at 85 months, with a maximum at 132 months. All the procedures of the study, including sample collection and study protocol, were in accordance with the Declaration of Helsinki and were approved by the Medical Ethics Committee of Yantai Affiliated Hospital of Binzhou Medical University (approval #: 20191127001), with an informed consent form waiver for the archived specimens.

### General Reagents

The general reagents for cell culture were purchased from Thermo Fisher Scientifics (Waltham, MA, USA). The chemicals used for protein expression were purchased from Takara, Inc. (Dalian, China), and the Nickel-His GraviTrap affinity column for protein purification was purchased from GE Healthcare. All the other chemicals were purchased from Sinopharm Chemicals (Beijing, China).

Rabbit anti-cyclinD1 monoclonal antibody (EP12) was purchased from ZSGB-BIO (Beijing, China). Horseradish peroxidase-labeled donkey anti-rabbit IgG secondary antibody was purchased from Jackson Immunoresearch lab (Pike West Grove, PA, USA). The Pierce bicinchoninic acid (BCA) protein quantification kit was purchased from Thermo Fisher Scientific, Inc. (Carlsbad, CA, USA). The recombinant human cyclinD1 protein was prepared in-house. The QDB plate was manufactured by Quanticision Diagnostics Inc., at RTP, NC, USA.

### Preparation of Recombinant cyclinD1 Protein

A DNA sequence corresponding to the 271-295AA of human cyclinD1 (NCBI #: NP_444284.1) was inserted into PET32a (+) expression vector and verified by DNA sequencing. The plasmid was expressed in BL21 (DE3) competent cells after the induction of isopropyl β-D-1-thiogalactopyranoside. Total bacterial protein lysate was extracted in binding buffer (20 mM sodium phosphate, 500 mM NaCl, 20 mM imidazole, pH 7.4) by ultrasonication. The recombinant cyclinD1 protein of bacterial lysate was purified by Ni-NTA column according to the instructions. Fractions containing cyclinD1 protein were pooled and concentrated on a Millipore ultrafiltration spin column. The protein concentration of cyclinD1 was determined using a BCA protein quantification kit, and the purity was determined by SDS-PAGE before subjecting to formalin fixation. The purified cyclinD1 protein was subject to fixation by incubating with nine volumes of 10% neutral buffered formalin solution at room temperature for 5 h before it was precipitated by nine volumes of cold acetone at -20°C for at least 2 h, followed by centrifugation for 10 mins at 12,000*g* to collect protein pellet. The pellet was washed once with ice cold acetone to remove residual formalin. The protein pellet was then dried at room temperature for 2 h before it was solubilized with a lysis buffer. The final protein concentration was determined using a BCA protein quantification kit.

### IHC Analysis

The IHC analyses for both Ki67 and cyclinD1 were performed using a procedure described elsewhere (8), using SA38-08 antibody for cyclinD1 and SP6 for Ki67 from LBP (www.gzlbp.com) at 1:100 dilution, respectively.

### Preparation of FFPE Tissues and Cell Lysates

Breast cancer FFPE specimens in 2 × 15-µm slices were de-paraffinized and solubilized with lysis buffer (50 mM HEPES, 137 mM NaCl, 5 mM EDTA, 1 mM MgCl_2_, 10 mM Na_2_P_2_O_7_, 1% TritonX-100, 10% glycerol). The MCF-7 cell pellet was collected and fixed in formalin for 30mins before solubilization with the same lysis buffer. The supernatants were collected after centrifugation for QDB analysis. The protein concentration was detected using a BCA protein quantification kit.

### QDB Analysis

The QDB process was described previously, with minor modifications (9–12). In brief, FFPE tissue lysate (about 0.35 μg/unit) and MCF-7 cell lysate (about 0.08 μg/unit) were applied onto the QDB plate, at 2 μl/unit, in triplicate. Serially diluted cyclinD1 recombinant protein was included in each plate as protein standard for the calculation of absolute cyclinD1 protein levels. The loaded QDB plate was dried in the air for 4 h and then blocked with a blocking buffer (4% non-fat milk in tris-buffered saline–Tween 20 for 1 h. The plate was inserted into a 96-well microplate pre-filled with 100 μl of anti-cyclinD1 antibody (clone EP12, 1:500 in blocking buffer) and incubated overnight at 4°C. The plate was rinsed twice and washed 4 × 10 mins before it was incubated with a donkey anti-rabbit secondary antibody for 4 h at room temperature. The plate was then rinsed twice and washed 5 × 10 mins before being processed with enhanced chemiluminescence reagent and quantified with a Tecan Microplate reader. The chemiluminescence signals were converted into absolute cyclinD1 protein level in each sample using a protein standard. All results were averages of three independent experiments, with each sample in triplicate.

MCF-7 cell lysate was used as internal control. The absolute cyclinD1 level of MCF-7 cell lysate was measured with the QDB method, and the results were documented. The overall experiment was judged as valid when the measured cyclinD1 level of MCF-7 cell lysate fell within ±20% of the documented value.

### Statistical Analysis

All statistical analyses were performed with either R version 3.6.2 or GraphPad Prism 7.0 software, using a two-sided statistical test. The results were reported as mean ± standard deviation (SD). A *p*-value of <0.05 was defined as statistically significant. All the missing values were treated as a new category. The cases lost to follow-up were not included in the analysis. Patients still alive at the last study follow-up were censored. The endpoint of overall survival analysis was defined as the time between breast surgery and death or last follow-up (April 1, 2019).

The correlation between cyclinD1 and Ki67 protein levels was analyzed with Spearman’s correlation coefficient analysis. Univariate Cox proportional hazard model-fitted overall survival was employed for hazard ratio (HR) and corresponding 95% confidence interval (CI) estimation. Multivariable Cox model was used to examine the association between Ki67 and/or cyclinD1 and OS adjusted for clinical factors, such as age, nodal positivity, pathological tumor size, tumor grade, and types of treatment. The Schoenfeld residuals test is used to test the proportional hazard assumption in the Cox model.

The cyclinD1 levels measured by the QDB method were dichotomized for OS analysis using an optimized cutoff value determined by the “surv_cutpoint” function of the “surviminer” R package.

cyclinD1 Harrell’s C-index was used as a measure of predictive accuracy of survival outcome. A C-index of 0.5 indicates an accuracy similar to random guessing and that of 1.0 indicates 100% predictive accuracy. We calculated unbiased estimates of the C-index for four combinations of variables to assess the contribution of different factors. Ki67 and cyclinD1 were used as categorical variables. The relationship between various subtypes or subgroups and OS of Luminal-like specimens was evaluated using the log-rank test and visualized by Kaplan–Meier survival curve analysis. The Schoenfeld residuals test is used to test the proportional hazard assumption in the Cox model.

## Results

### Absolute Quantitation of cyclinD1 Levels in 143 Luminal-Like Breast Cancer Specimens

**Figure 1:**
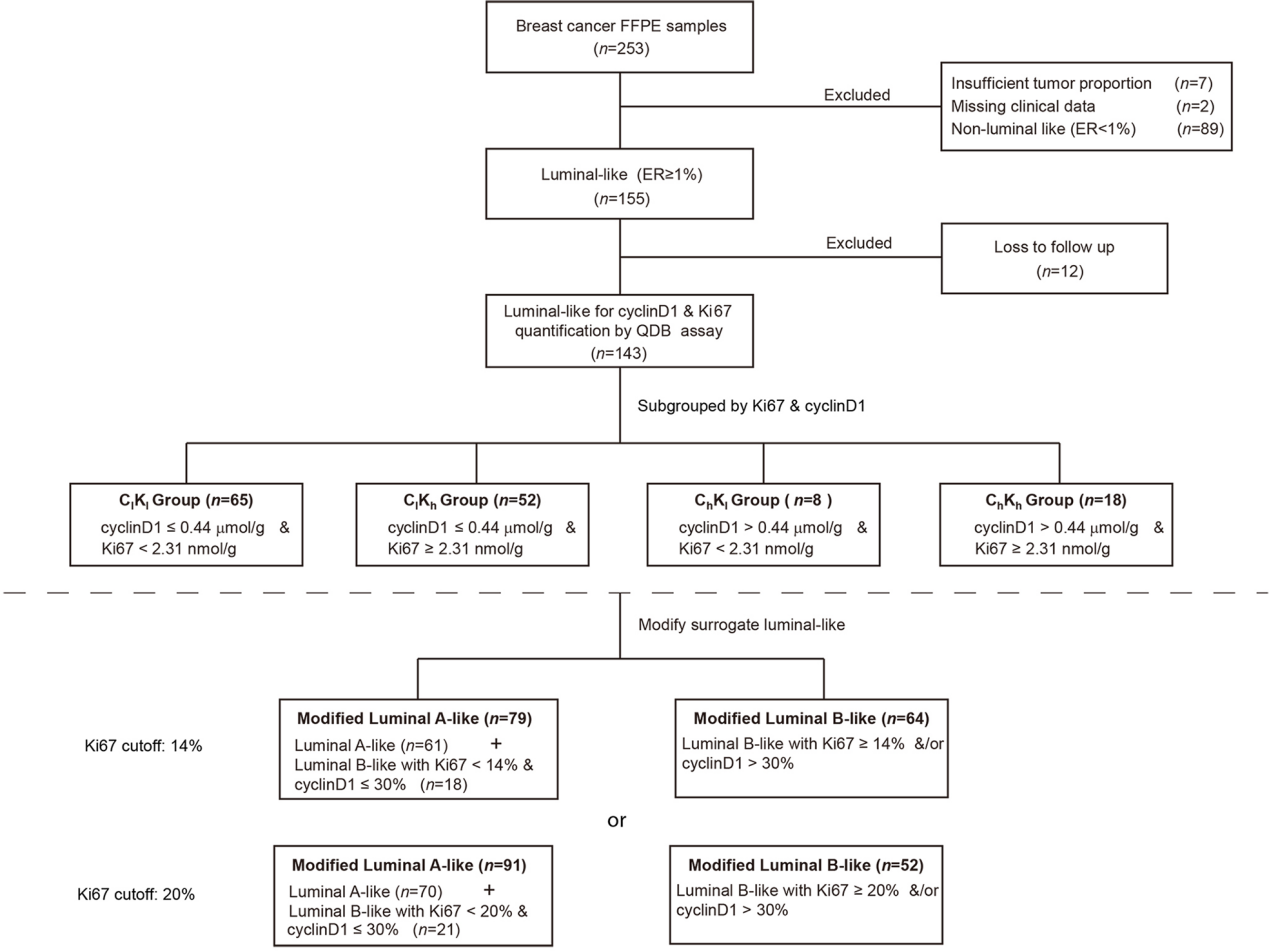
Flow diagram.

The clinicopathological characteristics of all 143 Luminal-like FFPE specimens with follow-up data are provided in **Table 1**. The flow chart of the specimens is shown in **Figure 1**. These Luminal-like specimens were collected sequentially and non-selectively from a local hospital, as described in the “Materials and Methods” section, with the median time to censoring at 85 months and maximum at 132 months.

**Table 1:** Clinicopathological characteristics of 143 Luminal-like specimens.

Characteristics	Number of cases (%)
Age	
Median	53
Range	31–82
Treatment type	
Endo^a^	2 (1.4)
Chemo^b^	85 (59.5)
Endo&Chemo^c^	36 (25.1)
Other^d^	20 (14.0)
Pathological lymph node status, pN	
pN0	67 (46.8)
pN1	56 (39.2)
pN2	9 (6.3)
pN3	7 (4.9)
Unknown	4 (2.8)
Pathological tumor size, pT	
pT1	50 (35.0)
pT2	83 (58.0)
pT3	7 (4.9)
Unknown	3 (2.1)
Histological grade	
II	78 (54.5)
III	48 (33.6)
Not applicable	17 (11.9)

The treatment plan was developed by physicians by following the guidance issued by the Chinese Anti-cancer Association in 2007 (13) at the discretion of the physician.

a (1) Tamoxifen or toremifene citrate tablet.

b (2) CAF (cyclophosphamide, doxorubicin hydrochloride, and fluorouracil) or CMF (cyclophosphamide, methotrexate, and fluorouracil) or TAC (doxorubicin hydrochloride and cyclophosphamide with or followed by docetaxel).

c One regimen from (1), followed by one regimen from (2).

d Non-standard treatments including Chinese traditional medicine or informed refusal by patients.

**Figure 2:**
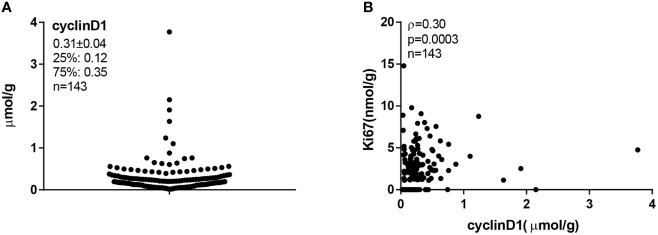
The cyclinD1 protein levels in 143 Luminal-like breast cancer formalin-fixed paraffin-embedded (FFPE) specimens. A total of 143 Luminal-like breast cancer FFPE tissues provided as 2 × 15-μm slices were collected sequentially and nonselectively from a local hospital, and the total protein lysates were prepared as described in “Materials and Methods”. MCF-7 cell lysate was used as internal control. FFPE tissue lysates (about 0.35 μg/unit) and cell lysate (about 0.08 μg/unit) were applied onto the Quantitative Dot Blot (QDB) plate at 2 μl/unit in triplicate for QDB analysis using anti-cyclinD1 antibody EP12. Serially diluted cyclinD1 recombinant protein was included in each plate as protein standard. All results were averages of three independent experiments, with each sample in triplicate. **(A)** The cyclinD1 levels were from 0.02 to 3.77 μmol/g, with a mean at 0.31 μmol/g and a median at 0.21 μmol/g. **(B)** The correlation between cyclinD1 and Ki67 protein levels in 143 FFPE tissues was analyzed with Spearman’s correlation coefficient analysis using Graphpad software, *ρ* = 0.30, *p* = 0.0003.

Total protein lysates were extracted from 2 × 15-μm FFPE tissue slices from each individual specimen. The linear ranges of both total tissue lysates and recombinant cyclinD1 protein were defined using the QDB method (**Supplementary Figure S1**). The distribution of cyclinD1 levels was from 0.02 to 3.77 μmol/g, with mean at 0.31 ± 0.04 μmol/g (**Figure 2A**). The expression levels of cyclinD1 were weakly correlated with those of Ki67 when analyzed using Spearman’s correlation analysis (*ρ* = 0.30, *p* = 0.0003, *n* = 143) (**Figure 2B**).

### cyclinD1 Was an Independent Prognostic Factor From Ki67

The putative prognostic role of cyclinD1, alongside Ki67, for OS of Luminal-like breast tumors was explored first with univariate survival analysis (**Table 2**). Both cyclinD1 and Ki67 were found to be independent prognostic factors for the OS of the patients, with HR for Ki67 at 1.17 (95% CI: 1.05–1.30, *p* = 0.0054) and for cyclinD1 at 2.33 (95% CI: 1.39–3.89, *p* = 0.0012), respectively. Meanwhile, the pathological node status and age were also found to be statistically significant.

**Table 2:** Univariate and multivariate Cox regression of overall survival (OS) of Ki67 and cyclinD1 values.

Variable	Univariate			Multivariate		
	HR	95% CI	*p*-value	HR	95% CI	*p*-value
Age	1.06	1.03–1.10	0.0004	1.07	1.04–1.11	0.0001
Treatment type^a^	1.14	0.84–1.54	0.4032	0.87	0.62–1.22	0.4139
Pathological lymph node status, pN	1.85	1.40–2.44	0.0001	1.80	1.28–2.53	0.0008
Pathological tumor Size, pT	1.39	0.82-2.35	0.2205	1.18	0.60–2.33	0.6366
Histological grade	1.09	0.65–1.83	0.7311	0.82	0.41–1.65	0.5780
Ki67	1.17	1.05–1.30	0.0054	1.16	1.01–1.33	0.0357
cyclinD1	2.33	1.39–3.89	0.0012	2.86	1.46–5.60	0.0022

Both the Ki67 and cyclinD1 levels of 143 Luminal-like patients were measured by quantitative dot blot analysis, and univariate and multivariate Cox regression analyses for OS were performed for these two sets of continuous data, respectively.

a A disordered categorical variable of endocrinotherapy, chemotherapy, endocrinotherapy and chemotherapy, and others to be assigned as 0, 1, 2, and 3, respectively, for statistical analysis.

These two biomarkers were further analyzed with multivariate Cox regression survival analysis (**Table 2**). Unexpectedly, although both factors were considered biomarkers of cell proliferation, they were found to be independent from each other, with HR for Ki67 at 1.16 (95% CI: 1.01–1.33, *p* = 0.0357) and for cyclinD1 at 2.86 (95% CI: 1.46–5.60, *p* = 0.0022). The age and pathological node status were other independent prognostic factors in the same analysis.

The contributions of both cyclinD1 and Ki67 to the OS of these patients were also evaluated using C-index (**Supplementary Figure S2**). The conventional clinical factors, including age, pathological tumor size, histological grade, and pathological node status, contributed collectively to the C-index at 0.72. The addition of Ki67 alone improved the C-index to 0.81, while the addition of cyclinD1 alone improved the C-index to 0.76. When both Ki67 and cyclinD1 were combined with conventional clinical factors, the C-index was improved to 0.82.

### Improving Prognosis Using Absolute Quantitated Ki67 and cyclinD1 Levels

Next, we attempted to apply these findings to improve the prognosis of Luminal-like tumors. An optimized cyclinD1 cutoff value was determined for OS analysis using the “surv_cutpoint” function of the “surviminer” R package at 0.44 μmol/g, and this value was used to separate these Luminal-like specimens into cyclinD1-high (C_h_) and cyclinD1-low (C_l_) groups. Meanwhile, these specimens were also separated into Ki67-high (K_h_) and Ki67-low (K_l_) groups using the 2.31 nmol/g defined in the previous study (8). In the accompanying manuscript III (submitted), the proposed 0.44 μmol/g cutoff was validated using an independent cohort from another hospital.

**Figure 3:**
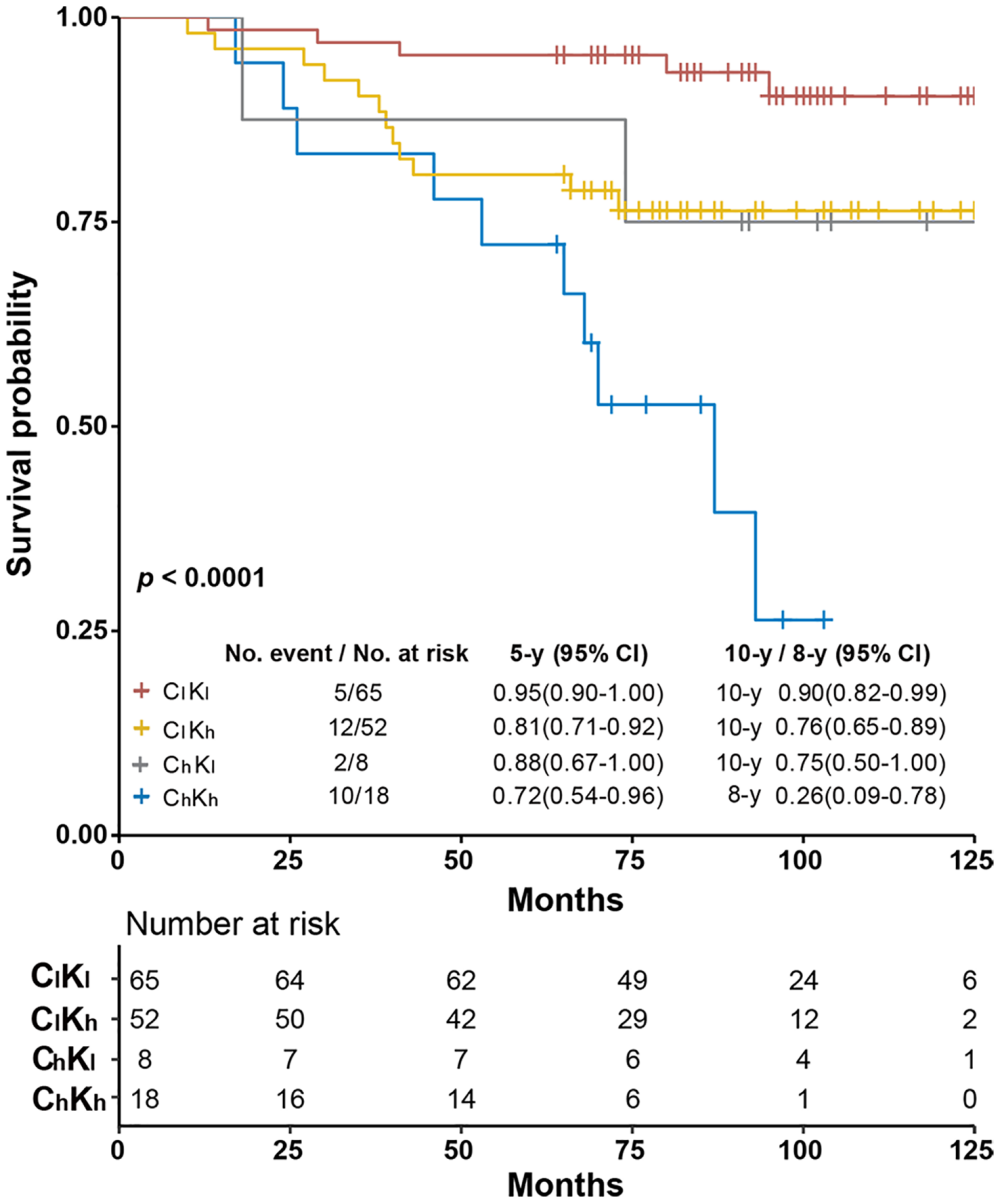
Kaplan–Meier survival analysis of overall survival of Luminal-like specimens subgrouped by objectively quantitated Ki67 and cyclinD1 protein levels. The specimens were separated into four subgroups using cyclinD1 at 0.44 μmol/g and Ki67 at 2.31 nmol/g as cutoffs, respectively. C_l_K_l_ stands for specimens with the protein levels of both biomarkers below the respective cutoffs, C_l_K_h_ for those with only cyclinD1 level below the recommended cutoff, C_h_K_l_ for those with only Ki67 levels below the recommended cutoff, and C_h_K_h_ for those with both biomarkers above the respective cutoffs. The 5- and 10-year survival probabilities and the *p*-values from log-rank test were provided in the figure.

These cutoffs were used together to separate these Luminal-like specimens into four subgroups. The C_h_K_h_ subgroup included specimens with both biomarkers above the respective cutoffs, while those in C_l_K_l_ were below the respective cutoffs. C_h_K_l_ referred to those with only cyclinD1 level above the corresponding cutoff, while the C_l_K_h_ referred to those with only Ki67 level above the corresponding cutoff. The OS of these four subgroups was analyzed using Kaplan–Meier survival analysis (**Figure 3**). We found that the C_l_K_l_ subgroup (*n* = 65) had a significantly improved 10y SP at 90% (95% CI: 0.82–0.99), while the C_h_K_h_ subgroup (*n* = 18) had the worst 8-year survival probability at 26% (95% CI: 0.09–0.78). The C_h_K_l_ (*n* = 8) and C_l_K_h_ (*n* = 52) subgroups had similar 10y SP at 75% (95% CI: 0.50–1.00) and 76% (95% CI: 0.65–0.89), respectively. The overall *p <*0.0001 from log-rank test. When compared with the C_l_K_l_ subgroup, the HR of the C_h_K_h_ subgroup increased by 9.02 folds (**Supplementary Figure S3**).

### Modifying Surrogate Assay Using cyclinD1 Levels Assessed by IHC Method

Our data suggested that the combined use of Ki67 and cyclinD1 from the QDB measurement may significantly improve the prognosis of Luminal-like patients. However, until protein biomarkers are routinely quantitated objectively in daily clinical practice, IHC analysis remains the primary method of biomarker assessment. Meanwhile, numerous Luminal-like breast cancer patients face the clinical decision of chemotherapy or no chemotherapy every day. Therefore, we explored the feasibility of applying our finding into daily clinical use by improving the performance of the surrogate assay using IHC-based cyclinD1 scores.

cyclinD1 levels from QDB measurements were plotted against the corresponding IHC scores, with the proposed 0.44 μmol/g shown in the plot (**Supplementary Figure S4**). We observed that the majority of the specimens with cyclinD1 scores ≤30% had their absolute levels <0.44 μmol/g. Thus, an IHC-based cyclinD1 score of 30% was used as putative cutoff to evaluate its impact on the performance of the surrogate assay.

**Figure 4:**
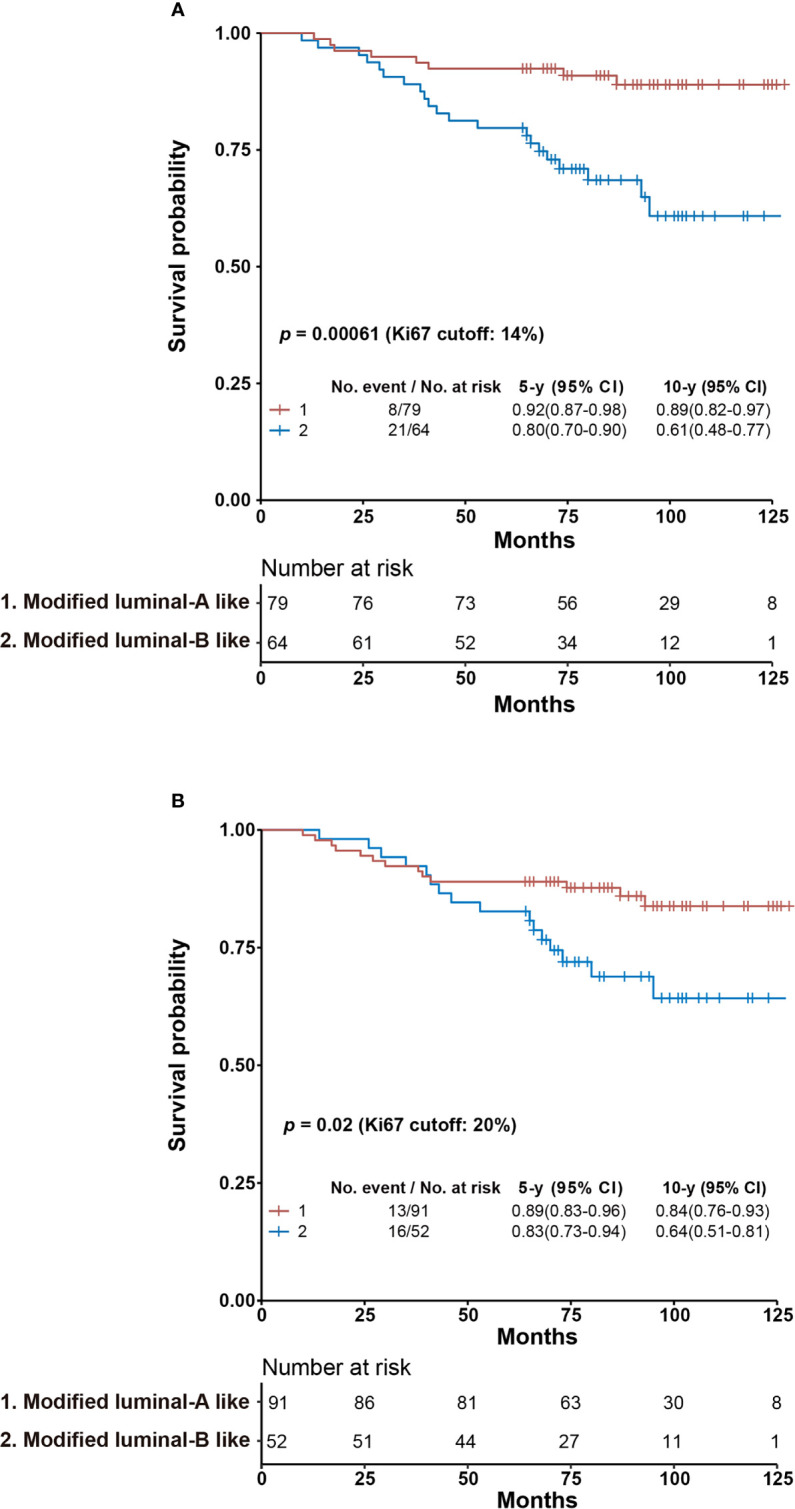
Kaplan–Meier overall survival analysis of modified Luminal A-like and Luminal B-like subtypes using Ki67 scores of 14% **(A)** or 20% **(B)** as cutoff. **(A)** Specimens with Ki67 <14% and cyclinD1 ≤30% from the Luminal B-like subtype based on surrogate assay using a Ki67 score of 14% as cutoff were combined with those of Luminal A-like subtype to become modified Luminal A-like subtype, while the rest of the Luminal B-like subtype (Ki67 <14% and cyclinD1 >30% or Ki67 ≥14%) was named as modified Luminal B-like subtype. **(B)** Specimens with Ki67 <20% and cyclinD1 ≤30% from Luminal B-like subtype based on surrogate assay using Ki67 score of 20% as cutoff were combined with those of Luminal A-like subtype to become modified Luminal A-like subtype, while the rest of the Luminal B-like subtype (Ki67 <20% and cyclinD1 >30% or Ki67 ≥20%) was named as modified Luminal B-like subtype. The survival probabilities of modified Luminal A-like and B-like subtypes were explored by Kaplan–Meier survival analysis with their 5- and 10-year survival probabilities and *p*-value from log-rank test as reported in the figure. CI, confidence interval.

The patients were stratified into Luminal A-like and Luminal B-like subtypes from the surrogate assay using an IHC-based Ki67 score of 14% and 20%, respectively. The stratification of Luminal A-like patients using cyclinD1 score of 30% as cutoff yielded no statistical difference using either a Ki67 score of 14% or 20% as cutoff. However, among the Luminal B-like subtype, we observed a significantly improved separation of those Ki67 <14% and cyclinD1 ≤ 30% from those of Ki67 ≥14% and/or cyclinD1 >30%, with *p* = 0.016 (**Supplementary Figure S5A**). We combined this subgroup (Ki67 <14% and cyclinD1 ≤ 30%) with the Luminal A-like subtype and named them modified Luminal A-like subtype. The rest of the Luminal B-like subtype (Ki67 <14 and cyclinD1 >30% or Ki67 ≥14%) were named as modified Luminal B-like subtype. We achieved 10y SP at 89% (*n* = 79) and 61% (*n* = 64) for the modified Luminal A subtype and B subtype, respectively, with *p* = 0.00061 from log-rank test. As comparison, the 10y SP for Luminal A-like subtype and Luminal B-like subtype was 88% (*n* = 61) and 68% (*n* = 82), respectively, with *p* = 0.031 from the surrogate assay using a Ki67 score of 14% as cutoff (**Figure 4A** and **Supplementary Table S1**).

When the Ki67 score of 20% was used as cutoff, we were unable to achieve statistical significance between those of Ki67 <20% and cyclinD1 ≤30% from those of Ki67 ≥20% and/or cyclinD1 >30% among Luminal B-like patients (**Supplementary Figure S5B**). Nonetheless, we assigned again the modified Luminal A-like subtype to include both patients of Ki67 <20% and cyclinD1 ≤30% of Luminal B-like subtype with those of Luminal A-like subtype. The rest of the Luminal B-like subtype (Ki67 <20% and cyclinD1 >30% or Ki67 ≥20%) were assigned as modified Luminal B-like subtype. We calculated their 10y SP at 84% for modified Luminal A-like subtype (*n* = 91) and 64% for modified Luminal B-like subtype (*n* = 52), with *p* = 0.02 using log-rank test. As comparison, the 10y SPs were 84% (*n* = 70) and 70% (*n* = 73) for Luminal A- and B-like subtypes, respectively, from the surrogate assay using a Ki67 score of 20% as cutoff, with *p* = 0.10 from log-rank test (**Figure 4B** and **Supplementary Table S1**).

## Discussion

In this study, cyclinD1 was demonstrated for the first time as an independent prognostic factor from Ki67 for the OS of Luminal-like patients. More importantly, the combined use of these two biomarkers was demonstrated to significantly improve the prognosis of Luminal-like patients. The subgroup with both biomarkers under the proposed cutoffs (C_l_K_l_) achieved best prognosis [10y SP: 90% (*n* = 65)], while those above the proposed cutoffs (C_h_K_h_) achieved the worst prognosis [8y SP: 26% (*n* = 18)]. This conclusion was validated using an independent cohort of Luminal-like patients to identified 107 patients out of 147 Luminal-like patients as C_l_K_l_ with 10y SP at 89% and six patients as C_h_K_h_ with 8y SP at 33% (see the accompanying manuscript Biomarker III). As a comparison, with the IHC-based surrogate assay, the Luminal A-like subtype and B-like subtype were found to have 10y SP at 88% (*n* = 61) and 68% (*n* = 82), respectively, when a Ki67 score of 14% was used as cutoff, and at 84% (*n* = 70) and 70% (*n* = 73) when a Ki67 score of 20% was used as cutoff (**Supplementary Table S1**).

Although extensive efforts have been devoted to explore the prognostic role of cyclinD1 among breast cancer patients, the conclusion remains controversial, even among patients of ER+. All these results were well summarized by Ahlin et al. and in several other studies (7, 14, 15). We suspect that these studies were seriously limited by the method that they used. Indeed IHC is used primarily for the assessment of cyclinD1 protein levels in breast cancer specimens (7), yet this method is known to be associated with inconsistency and subjectivity (16). The QDB method should circumvent these issues to reveal a strong correlation of cyclinD1 overexpression with the reduced survival of ER+ patients, supported by both the univariate and multivariate Cox regression survival analyses in this study.

We also attempted to apply these findings in the IHC-based surrogate assay, as IHC remains the predominant method in daily clinical practice worldwide. However, the inherent issues with IHC analysis, including the subjectivity and inconsistency of the method, cause considerable trouble with this effort (16, 17). As shown in **Supplementary Figure S4**, only at lower range of cyclinD1 score (≤30%) were we able to achieve a tolerable agreement between the QDB method and IHC analysis.

Nonetheless, our efforts suggested that the incorporation of cyclinD1 in daily clinical practice may potentially improve the performance of the surrogate assay. The number of patients with bad prognosis was reduced from 82 to 64, with 10y SP decreased from 68 to 61%, while those with good prognosis increased from 61 to 79, with 10y SP increased from 88 to 89%, when a Ki67 score of 14% was used as cutoff. When a Ki67 score of 20% was used as cutoff, the number of bad prognosis was reduced from 73 to 52, with 10y SP decreased from 70 to 64%, while those of good prognosis increased from 70 to 91, with 10y SP unchanged. These results suggested a potential clinical significance of cyclinD1 in daily clinical practice (**Supplementary Table S1**).

It should be pointed out that an IHC score of 30%, instead of 50%, was chosen as the cutoff for more applicability in daily practice, although in this study, the cyclinD1 level of almost all specimens with an IHC score of 50% was below the proposed 0.44 μmol/g cutoff (**Supplementary Figure S4**). Admittedly, this study was limited by the small sample size, with only the OS of these patients investigated. For this reason, the applicability of the suggested 30% cutoff from the IHC analysis remains to be tested in future studies. Nonetheless, we wish to convey the message that patients with high expression levels of both Ki67 and cyclinD1 face a significantly higher risk than those with low expression levels of both biomarkers. We encourage clinicians with access to large datasets to validate this finding and develop an optimized IHC-based cyclinD1 cutoff to be used in daily clinical practice.

It should also be emphasized that this study is by no means dismissing the prognostic role of PR (18), rather, this factor is yet to be incorporated in the current prognostic method. In fact, consistent with the protective role of PR in the surrogate assay, the only two deaths in the C_l_K_l_ subgroup in our study are with low expression of PR (unpublished data). A better algorithm is expected to be developed to incorporate PR, cyclinD1, and Ki67 in the prognosis evaluation of Luminal-like patients, with more specimens included in the study.

While this study suggested the potential usage of cyclinD1 to aid in the clinical diagnosis of Luminal-like breast cancer patients, it nonetheless was a retrospective observational study susceptible to bias. Thus, it should be considered a pilot study. A study of a much larger scale, possibly prospective, is needed to validate the usage of this biomarker in daily clinical practice.

The study was also negatively influenced by a large number of Luminal-like patients not receiving endocrine therapy. The treatments that the patients received in this study were developed based on guidance from the China Anti-cancer Association (CACA) issued in 2007 (13). The concept of subtyping breast cancer patients was not gaining popularity in China at that period, especially in a local hospital. This issue further emphasized the need of a prospective study to avoid potential biases inherently associated with a lot of retrospective clinical studies.

In conclusion, by measuring the cyclinD1 protein levels absolutely and quantitatively in 143 Luminal-like FFPE specimens, we showed that the overexpression of cyclinD1 was negatively associated with the survival of Luminal-like patients, and the combined use of cyclinD1 and Ki67 may significantly improve the prognosis of Luminal-like patients. This study supports the prospective investigation of cyclinD1 relevance in the clinic.

## Data Availability Statement

The original contributions presented in the study are included in the article/**Supplementary Material**. Further inquiries can be directed to the corresponding author.

## Ethics Statement

The studies involving human participants were reviewed and approved by the Ethics Committee of Yantai Affiliated Hospital of Binzhou Medical University (approval no. 20191127001). The Ethics Committee waived the requirement of written informed consent for participation.

## Author Contributions

JH, JRZ, SX, and CZ provided clinical samples. JH supervised all the clinical studies. WZ, YL, YZ, JL, and FT performed all the assays and data analysis. FT supervised all the assays. YL and JBZ performed all the statistical analysis. WZ, YL, and JDZ contributed to data interpretation and edited the manuscript. JDZ designed and supervised the overall study and drafted the manuscript. All authors contributed to the article and approved the submitted version.

## Funding

This research is funded by Quanticision Diagnostics, Inc. All sources of funding received for the research have been submitted.

## Conflict of Interest

WZ, YL, YZ, JL, JBZ, JDZ, and FT are employees of Yantai Quanticision Diagnostics, Inc., a division of Quanticision Diagnostics, Inc., who own or has filed patent applications for QDB plate, QDB method, and QDB application in clinical diagnostics.

The remaining authors declare that the research was conducted in the absence of any commercial or financial relationships that could be construed as a potential conflict of interest.

## Publisher’s Note

All claims expressed in this article are solely those of the authors and do not necessarily represent those of their affiliated organizations, or those of the publisher, the editors and the reviewers. Any product that may be evaluated in this article, or claim that may be made by its manufacturer, is not guaranteed or endorsed by the publisher.

## Abbreviations

QDB, quantitative dot blot method; FFPE, formalin-fixed paraffin-embedded; ER, estrogen receptor; PR, progesterone receptor; Her2, human epidermal growth factor receptor 2; IHC, immunohistochemistry; OS, overall survival; SP, survival probability; FISH, fluorescence *in situ* hybridization; SD, standard deviation.
