# Supplementary material for: Combined Use of cyclinD1 and Ki67 for Prognosis of Luminal-Like Breast Cancer Patients
Source: Front Oncol. 2021 Nov 9;11:737794. doi: 10.3389/fonc.2021.737794 (PMC8630735; doi:10.3389/fonc.2021.737794)
Supplement: Supplementary file 1 [file DataSheet_1.docx]

**Supplementary figures and tables**

**
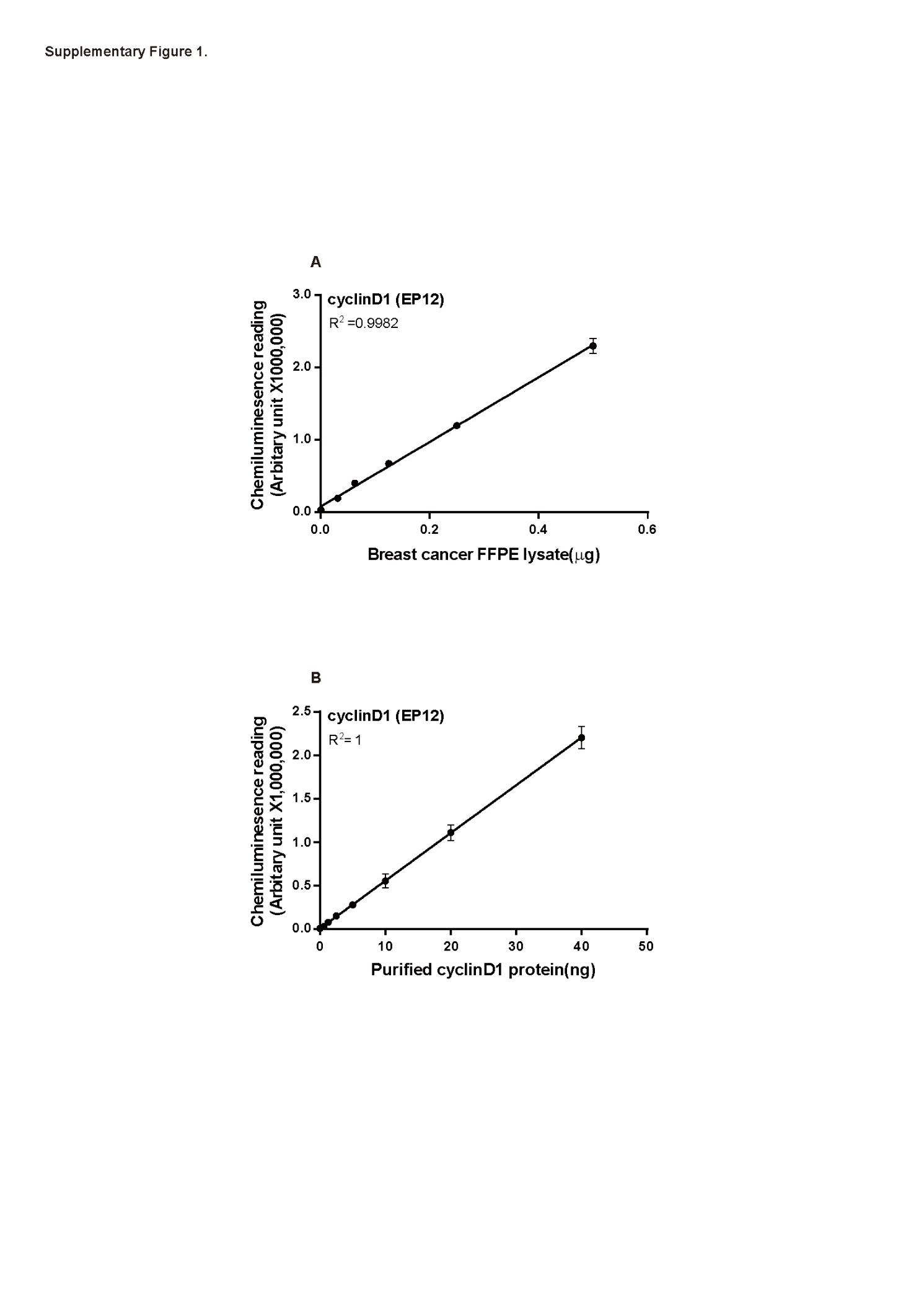
**
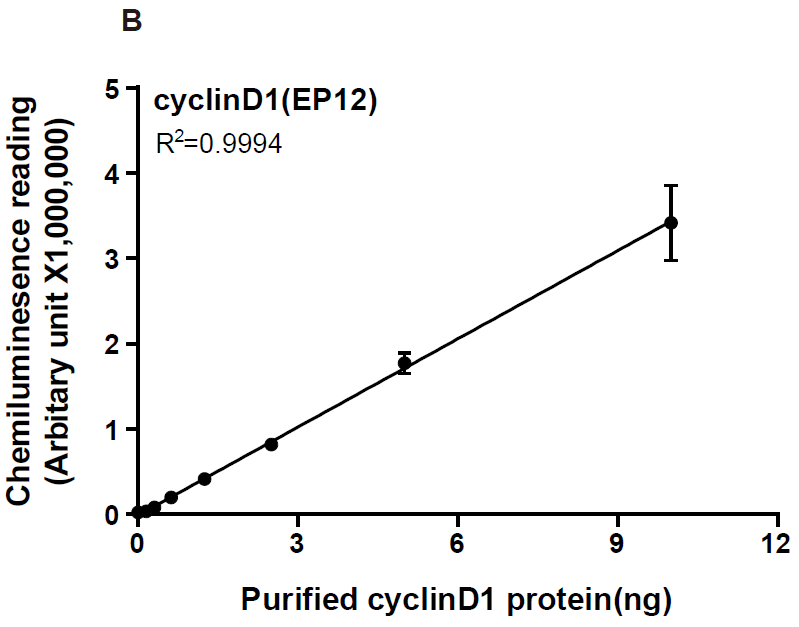


**Figure S1. Defining the linear ranges of QDB analysis. (A)** Defining the linear range of QDB method with breast cancer FFPE lysate. Human breast cancer FFPE specimens (2x15 μm slices) were provided sequentially and non-selectively by local hospital, and the pooled FFPE lysate was prepared by mixing in equal amount of the total tissue lysates prepared from 4 specimens of high IHC scores for cyclinD1. The pooled lysate was serially diluted as indicated in the figure and supplemented with IgG-Free BSA solution to allow for equal loading (about 0.5 μg/unit). The lysate was applied onto QDB plate at 2 μl/unit for QDB analysis using anti-cyclinD1 antibody (clone EP12). **(B)** Defining the linear range of QDB method with purified cyclinD1 protein. The cyclinD1 recombinant protein was serially diluted as indicated in the figure and supplemented with IgG-Free BSA solution. The diluted recombinant protein lysate was applied onto the QDB plate at 2 μl/unit for QDB analysis using anti-cyclinD1 antibody (clone EP12).

**
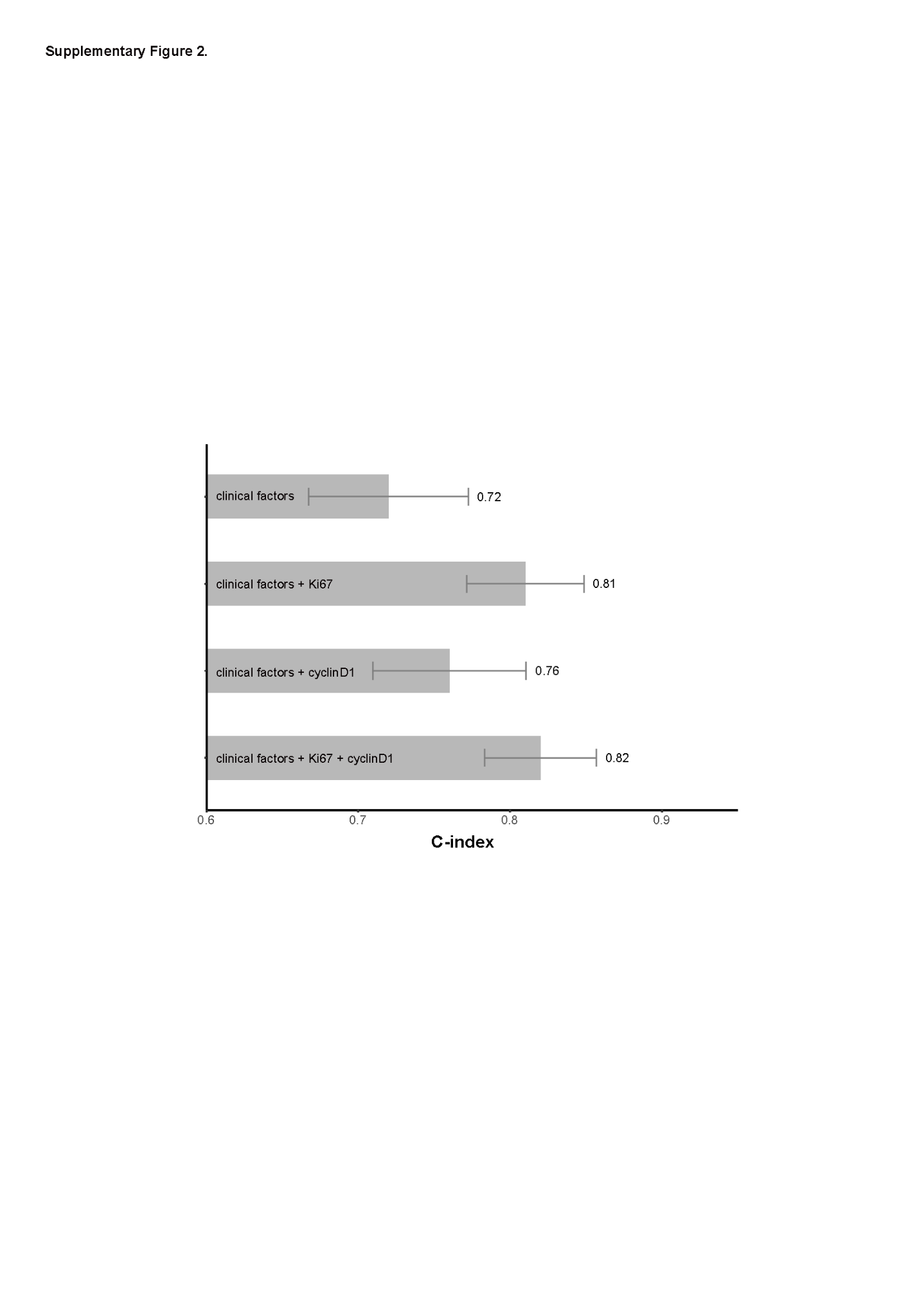
**

**Figure S2. C-index analyses to evaluate the contributions of cyclinD1, Ki67 and their combinations to OS.** Four models were compared for prediction of overall survival with clinical factors alone [age, pathological node status (pN), pathological tumor size (pT), histological grade, and type of treatment]; addition of absolutely quantitated Ki67 alone; addition of absolutely quantitated cyclinD1 alone; and addition of both biomarkers concurrently. The values on the x-axis were unbiased estimates of the c-index for different combinations of variables by Cox regression.


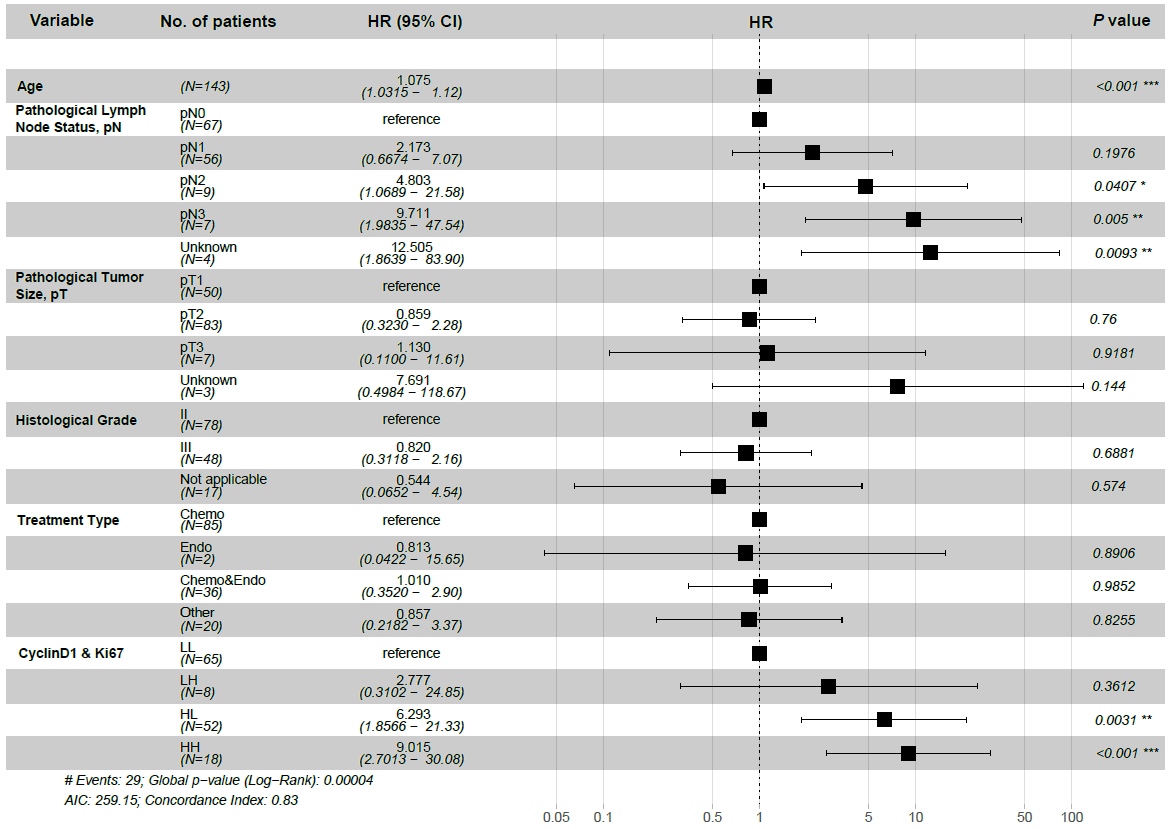


**Figure S3. Forest plot for OS analysis based on multivariate cox model.**  The forest plot showed the adjusted HRs and 95% CIs of several clinical factors and cyclinD1 and Ki67 in combination in 143 Luminal-like specimens. HR: Hazard Ratio; CI: Confident Interval.


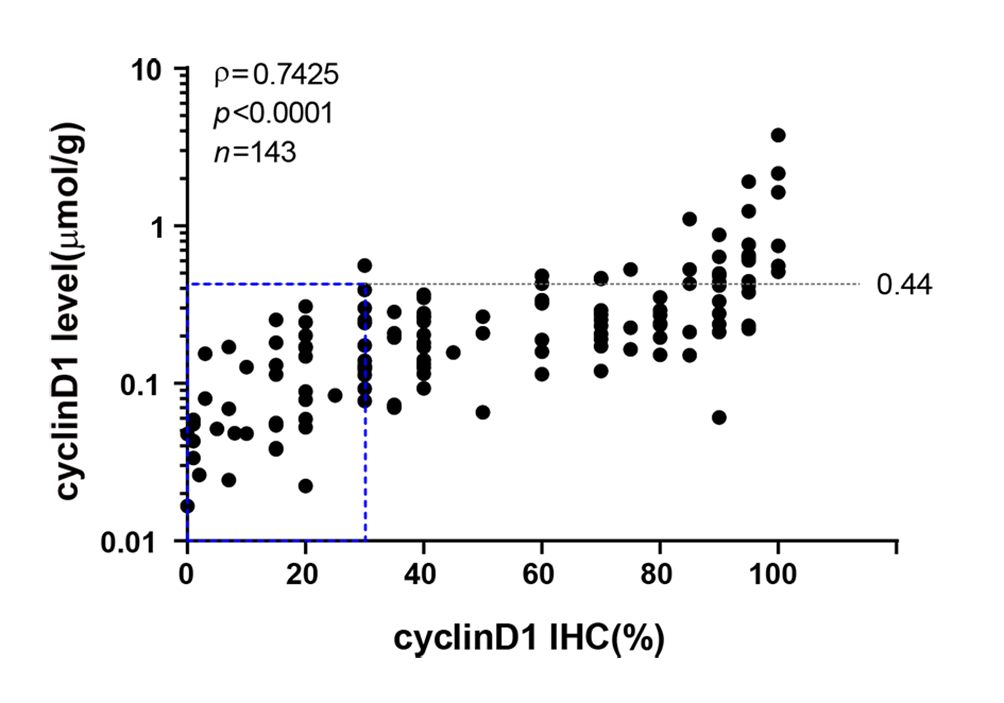


**Figure S4. Comparison of QDB results with IHC scores of cyclinD1.** ·The cyclinD1 levels from QDB analysis were plotted against the provided IHC results from local hospital, and the proposed cutoff at 0.44 μmole/g was shown as a dashed line in the plot. The correlation was analyzed with Spearman’s rank correlation analysis, with ρ=0.7425, p<0.0001. As shown in the figure, the majority of plots with cyclinD1 scores ≤30% had their QDB results <0.44 μmole/g, as indicated in the blue box.


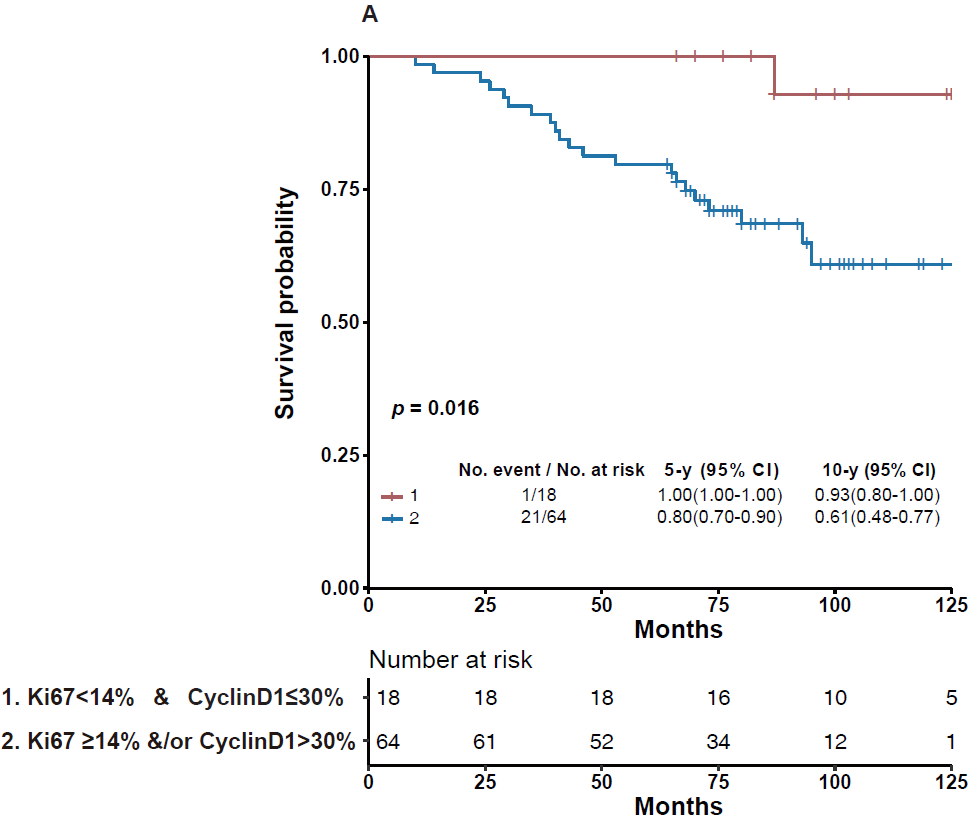

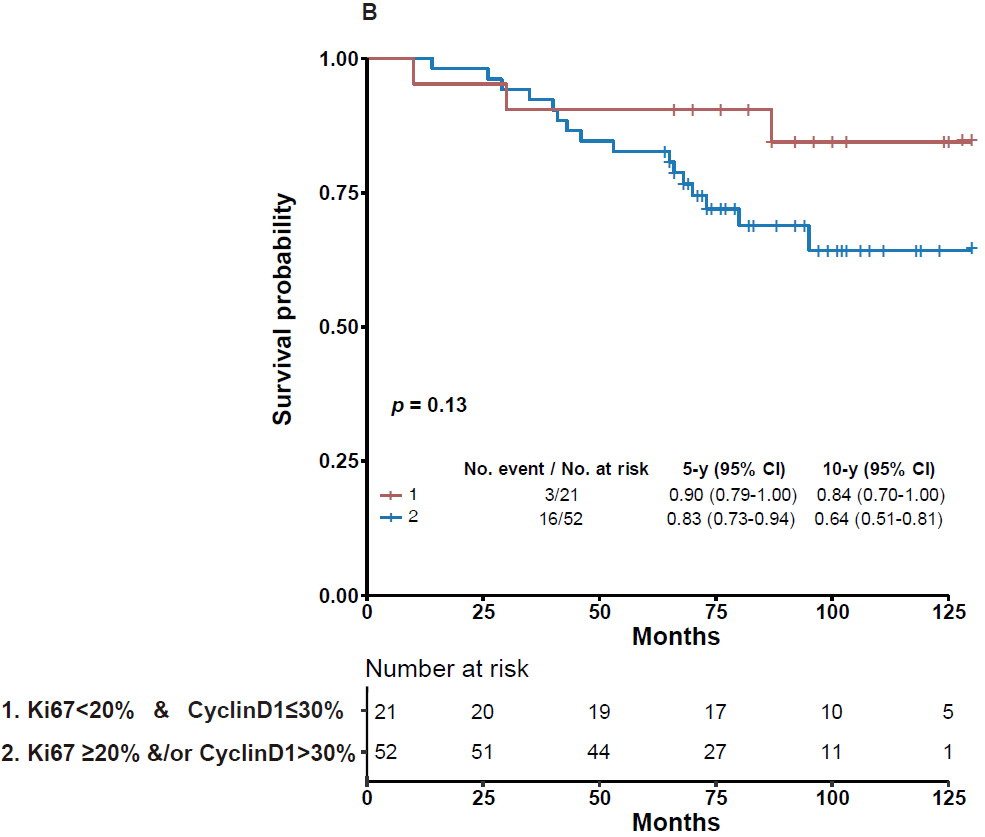


**Figure S5.** **Kaplan-Meier curves for OS of Luminal-B like specimens stratified into two subgroups using IHC-based cyclinD1 score of 30% as cutoff.** **(A)** Total of 82 Luminal B-like specimens from surrogate assay using Ki67 score of 14% as cutoff were further separated into two subgroups, with one subgroup as those with Ki67<14% & cyclinD1≤ 30%, and the other group as either Ki67<14% & cyclinD1>30%, or Ki67≥14%. The survival probabilities of these two sub-groups were analyzed with Kaplan-Meier survival analysis, with p=0.016 from Log Rank test. The 5-year and 10-year survival probabilities of these two subgroups were also shown in the figure. **(B)** Total of 73 Luminal B-like specimens from surrogate assay using Ki67 score of 20% as cutoff were further separated into two subgroups, with one subgroup as those with Ki67<20% & cyclinD1≤ 30%, and the other group as either Ki67<20% & cyclinD1>30%, or Ki67≥20%. The survival probabilities of these two sub-groups were analyzed with Kaplan-Meier survival analysis, with p=0.13 from Log Rank test. The 5-year and 10-year survival probabilities of these two subgroups were also shown in the figure.

| **Table S1. Performance of five subtyping methods** | | | |
| --- | --- | --- | --- |
| Methods | No. event / No. at risk(%) | OS probability (95% CI) | p-value |
| CyclinD1&Ki67 method^1^ |  |  | < 0.0001 |
| C_l_K_l_ | 5/65(7.7) | 10-y 0.90(0.82-0.99) |  |
| C_h_K_l_ | 2/8(25.0) | 10-y 0.75(0.50-1.00) |  |
| C_l_K_h_ | 12/52(23.1) | 10-y 0.76(0.65-0.89) |  |
| C_h_K_h_ | 10/18(55.6) | 8-y 0.26(0.09-0.78) |  |
| Modified surrogate assay^2^  (Ki67 cutoff: 14%) |  |  | 0.00061 |
| Luminal A_i_ | 8/79(10.1) | 10-y 0.89(0.82-0.97) |  |
| Luminal B_i_ | 21/64(32.8) | 10-y 0.61(0.48-0.77) |  |
| Surrogate assay[2]  (Ki67 cutoff: 14%) |  |  | 0.031 |
| Luminal A_i_ | 7/61(11.5) | 10-y 0.88(0.80-0.97) |  |
| Luminal B_i_ | 22/82(26.8) | 10-y 0.68(0.58-0.81) |  |
| Modified surrogate assay^3^ (Ki67 cutoff: 20%) |  |  | 0.02 |
| Luminal A_i_ | 13/91(14.3) | 10-y 0.84(0.76-0.93) |  |
| Luminal B_i_ | 16/52(30.8) | 10-y 0.64(0.51-0.81) |  |
| Surrogate assay[5]  (Ki67 cutoff: 20%) |  |  | 0.10 |
| Luminal A_i_ | 10/70(14.3) | 10-y 0.84(0.75-0.94) |  |
| Luminal B_i_ | 19/73(26.0) | 10-y 0.70(0.60-0.83) |  |
| **^1^**: C_l_K_l_: specimens with both cyclinD1 and Ki67 levels below suggested cutoffs; C_h_K_l_: specimens with only Ki67 levels below the suggested cutoff; C_l_K_h_: specimens with only cyclinD1 below the suggested cutoff; C_h_K_h_: specimens with both cyclinD1 and Ki67 levels above the suggested cutoffs.  ^2^: Modified Luminal A_i_: Luminal A_i_, and Ki67<14% & cyclinD1≤ 30% fraction of Luminal B_i_ from surrogate assay;  Modified Luminal B_i_: Ki67<14% & cyclinD1>30%, or Ki67≥14% from Luminal B_i_ of Surrogate assay.  ^3^: Modified Luminal A_i_: Luminal A_i_, and Ki67<20% & cyclinD1≤ 30% fraction of Luminal B_i_ from surrogate assay; Modified Luminal B_i_: Ki67<20% & cyclinD1>30%, or Ki67≥20% from Luminal B_i_ of Surrogate assay. | | | |
